# *Francisella tularensis* Outer Membrane Vesicles Participate in the Early Phase of Interaction With Macrophages

**DOI:** 10.3389/fmicb.2021.748706

**Published:** 2021-10-15

**Authors:** Ivona Pavkova, Jana Klimentova, Jan Bavlovic, Lenka Horcickova, Klara Kubelkova, Erik Vlcak, Helena Raabova, Vlada Filimonenko, Ondrej Ballek, Jiri Stulik

**Affiliations:** ^1^Department of Molecular Pathology and Biology, Faculty of Military Health Sciences, University of Defence, Hradec Kralove, Czechia; ^2^Electron Microscopy Core Facility, Institute of Molecular Genetics of the Czech Academy of Sciences, Prague, Czechia; ^3^Department of Biology of the Cell Nucleus, Institute of Molecular Genetics of the Czech Academy of Sciences, Prague, Czechia; ^4^Laboratory of Immunobiology, Institute of Molecular Genetics of the Czech Academy of Sciences, Prague, Czechia

**Keywords:** *Francisella tularensis*, FSC200, outer membrane vesicles, host–pathogen interaction, macrophage, cell entry

## Abstract

*Francisella tularensis* is known to release unusually shaped tubular outer membrane vesicles (OMV) containing a number of previously identified virulence factors and immunomodulatory proteins. In this study, we present that OMV isolated from the *F. tularensis* subsp. *holarctica* strain FSC200 enter readily into primary bone marrow-derived macrophages (BMDM) and seem to reside in structures resembling late endosomes in the later intervals. The isolated OMV enter BMDM generally *via* macropinocytosis and clathrin-dependent endocytosis, with a minor role played by lipid raft-dependent endocytosis. OMVs proved to be non-toxic and had no negative impact on the viability of BMDM. Unlike the parent bacterium itself, isolated OMV induced massive and dose-dependent proinflammatory responses in BMDM. Using transmission electron microscopy, we also evaluated OMV release from the bacterial surface during several stages of the interaction of *Francisella* with BMDM. During adherence and the early phase of the uptake of bacteria, we observed numerous tubular OMV-like protrusions bulging from the bacteria in close proximity to the macrophage plasma membrane. This suggests a possible role of OMV in the entry of bacteria into host cells. On the contrary, the OMV release from the bacterial surface during its cytosolic phase was negligible. We propose that OMV play some role in the extracellular phase of the interaction of *Francisella* with the host and that they are involved in the entry mechanism of the bacteria into macrophages.

## Introduction

Release of extracellular vesicles is a highly conserved and natural process known in all kingdoms of life: in Archaea, Bacteria, and Eukarya (Gill et al., 2018). Regarding bacteria, the vesicles are known to be involved in multiple situations during their life cycle. Generally, the vesicle release serves the bacteria for interaction with the environment, for communication with other bacteria in their community, and in the case of pathogenic bacteria, also for interaction with the host (Ellis and Kuehn, 2010; Avila-Calderón et al., 2014; Jan, 2017). Vesicles of Gram-negative bacteria are derived from their outer membrane, thus are termed outer membrane vesicles (OMV), and they are formed by the bulging of the membrane and closing inside a portion of the periplasm (Schwechheimer and Kuehn, 2015). Being derived from the bacterial envelope, OMV are highly enriched in such immunostimulatory materials as the outer membrane proteins, lipids, lipoproteins, glycoproteins, and lipopolysaccharide. The cargo of pathogenic bacteria OMV also often contains virulence factors, toxins, or other biologically active molecules that play important roles in host–pathogen interaction. OMV are known to enhance the invasiveness and survival of bacteria inside the host (Jan, 2017). OMV can increase the bacterial adherence to various surfaces (Ellis and Kuehn, 2010), they are part of the membrane stress response to hostile environments (MacDonald and Kuehn, 2013; Klimentova et al., 2019), and they can modulate the host immune response (Kaparakis-Liaskos and Ferrero, 2015). Great attention is currently focused on the vaccine potential of OMV because of their strong immunomodulatory effect, self-adjuvant characteristic, and the ease of genetic modifications (Acevedo et al., 2014; Bitto and Kaparakis-Liaskos, 2017; Yang et al., 2018; Gerritzen et al., 2019).

The diverse fates of OMV released from extracellular bacteria inside the host encompass interaction with the extracellular matrix or direct effects on the host cells. The latter proceed after binding to one of the host surface structures, either by membrane fusion followed by the release of the vesicle’s inner content directly into the host cell cytosol or by the vesicle’s internalization. The internalization of OMV may proceed *via* various pathways, and their further intracellular trafficking depends upon the type of vacuole within which they end up. OMV engulfment by immune cells is also the explanation for lipopolysaccharide (LPS) intracellular sensing and the non-canonical inflammasome activation observed in extracellular bacteria (Vanaja et al., 2016). Much less is known about the OMV secretion from intracellularly surviving bacteria during their intracellular phase. The secretion of OMV inside macrophages has been proven by electron microscopy in *Salmonella* (Yoon et al., 2011), *Listeria* (Vdovikova et al., 2017), and *Mycobacteria* (Prados-Rosales et al., 2011; Athman et al., 2015). In *Legionella pneumophila*, OMV have been found to inhibit the phagosome–lysosome fusion and to interrupt phagosome maturation in order to allow the intracellular survival and replication of bacteria (Fernandez-Moreira et al., 2006).

*Francisella tularensis*, a Gram-negative facultative intracellular bacterium, is one of the most infectious bacteria and is a potential biological warfare agent (Dennis et al., 2001; Oyston, 2008). The extremely high virulence of *Francisella* is caused by its ability to invade and proliferate inside a range of host cell types—macrophages and dendritic cells being its primary target cells (Celli and Zahrt, 2013)—and its ability to successfully overcome innate immune mechanisms (Putzova et al., 2017; Fabrik et al., 2018). After internalization, *Francisella* resides in a phagosomal vacuole, the maturation to phagolysosome of which is arrested by a yet unknown mechanism, it escapes rapidly into the cytosol, and there it proliferates (Clemens and Horwitz, 2007; Celli and Zahrt, 2013; Ozanic et al., 2015). *Francisella* spp. have been described to produce OMV of tubular shape that contain a number of virulence factors and take part in bacterial stress response (McCaig et al., 2013; Sampath et al., 2018; Klimentova et al., 2019, 2021). Similar membrane nanotubes with adhesive and communication functions have been described in *Salmonella typhimurium* (Galkina et al., 2011). In *Francisella novicida*, which is a close relative to *F. tularensis* but displays very low virulence in human, the formation of tubular outer membrane protrusions has been shown to occur in close vicinity to the macrophage cytoplasmic membrane in the early stage of interaction (McCaig et al., 2013).

In the present study, we focused on the effects on the host cells of OMV released by the virulent *F. tularensis* subsp. *holarctica* strain FSC200. We describe here the entry of isolated and purified OMV into the host cells with a view also to their further intracellular fate. The vesicles proved to be non-toxic *in vitro* to primary macrophages, but they triggered a strong proinflammatory response that is in contrast with the ability of the bacterium itself to efficiently inhibit the immune pathways. We also documented OMV release from the parent bacteria in contact with the host cells during the early stage of the bacterial internalization by macrophages. On the contrary, we were not able to prove OMV release from the bacterial surface during its cytosolic phase. Our findings nevertheless point to the engagement of *Francisella* OMV in the pathogenesis of the bacterium and their future potential in the research of protective agents.

## Materials and Methods

### Bacterial Strains and Cultivation

*Francisella tularensis* subsp. *holarctica* strain FSC200 (Johansson et al., 2000) was kindly provided by Åke Forsberg (Swedish Defence Research Agency, Umeå, Sweden). FSC200 with inactivated production of O-antigen (FSC200/Δ*wbtDEF*) from an earlier study (Balonova et al., 2012) was used here. Stock bacteria were pre-cultivated on McLeod agar supplemented with bovine hemoglobin and IsoVitaleX (Becton Dickinson, Le Pont de Claix, France) at 37°C for 24 h. Brain heart infusion (BHI; Becton Dickinson) was prepared according to the manufacturer’s instructions. pH was adjusted with HCl to 6.8, and it was sterile filtered instead of autoclaved.

### Outer Membrane Vesicles Isolation and Purification

Outer membrane vesicles were prepared as described earlier (Klimentova et al., 2019). Briefly, bacteria from agar plate were inoculated to BHI and pre-cultivated for 10–14 h at 37°C and 200 rpm. Starting cultures were pelleted (6,000 × *g*, 15 min at 25°C), diluted with fresh medium to OD_600_ = 0.1, and then cultivated for 14–16 h [final optical density (OD) of approx. 0.6]. Bacteria were removed by low-speed centrifugation and the supernatants sterilized by filtration through a 0.22-μm vacuum-driven filter. Culture filtrates were concentrated using an Amicon^®^ Stirred Ultrafiltration Cell through membrane of regenerated cellulose with 100 kDa cutoff (both Millipore, Billerica, MA, United States) and subsequently pelleted (100,000 × *g*, 90 min at 4°C). The pellet was resuspended in 45% (*w*/*v*) OptiPrep (Sigma-Aldrich, St. Louis, MO, United States) in 10 mM HEPES/0.85% NaCl, pH 7.4 (HEPES buffer), and overlaid with a step OptiPrep gradient of 40%–20%. The gradient was centrifuged at 100,000 × *g* for 16–20 h at 4°C in a swinging-bucket rotor. After centrifugation, the top fractions containing a clearly visible opaque white band were carefully collected, diluted 8× with HEPES buffer, and centrifuged (100,000 × *g*, 2 h at 4°C). The supernatant was removed and the pellet washed again (same conditions) to remove the residual OptiPrep. The final pellet was suspended in physiological saline and the protein concentration determined with Micro BCA^TM^ Protein Assay Kit (Pierce, Rockford, IL, United States). OMV samples were stored at +4°C for up to 2 weeks.

### Cell Culture

The human alveolar type II epithelial cell line A549 (ATCC^®^ CCL-185^TM^) and the mouse BALB/c monocyte–macrophage cell line J774.2 (Merck, Darmstadt, Germany) were cultured in Dulbecco’s modified Eagle’s medium (DMEM) supplemented with 10% (*v*/*v*) fetal bovine serum (FBS; United States origin, Sigma-Aldrich) at 37°C in 5% CO_2_.

Bone marrow-derived macrophages (BMDM) were generated from the femurs and tibias of 6- to 10-week-old female BALB/c mice as described previously (Celli, 2008). Briefly, cells flushed from the bone marrow were placed in bacteriological Petri dishes and differentiated in DMEM supplemented with 10% FBS, 20% (*v*/*v*) L929-conditioned medium (as a source of macrophage colony-stimulating factor), and 50 U/ml penicillin/50 μg/ml streptomycin (only for the first 3 days of cultivation). After 6 days of differentiation, the BMDM were seeded on tissue culture-treated multiwell plates at the desired density as further specified in the relevant assay procedure.

### Outer Membrane Vesicles Internalization Assays

To detect the ability of OMV to invade host cells, BMDM, J774.2, or A549 cells were seeded onto sterile glass coverslips in 24-well plates (at a concentration of 1 × 10^5^ cells/well) and treated with OMV (0.5 μg) for 10 min or for 1, 4, or 24 h. At indicated times, the cells were fixed in 3.7% paraformaldehyde (PFA; pH 7.2) for 10 min at room temperature (RT), washed three times with phosphate-buffered saline (PBS), quenched with 50 mM ammonium chloride for 10 min at RT, and then washed three times with PBS. The cells were next permeabilized with 0.1% Triton X-100 for 5 min at RT and blocked with 3% (*w*/*v*) bovine serum albumin (BSA) in PBS for 60 min at RT. To label the internalized OMV, the cells were first incubated with purified immune rabbit polyclonal anti-*F. tularensis* serum and then, after four washes with PBS, with Alexa Fluor^TM^ 488 anti-rabbit immunoglobulin G (IgG; Life Technologies, Carlsbad, CA, United States). Actin was labeled with phalloidin-TRITC (Sigma-Aldrich) and nuclei with DAPI (Invitrogen, Waltham, MA, United States) according to the manufacturers’ instructions. After three washes with PBS and one wash with deionized water, the coverslips were mounted on glass slides with ProLong Diamond Antifade Mountant (Invitrogen) and imaged on a Nikon Eclipse Ti fluorescence microscope equipped with a Plan-Apochromat ×60/1.4 oil immersion objective (Nikon, Tokyo, Japan) and the NIS Elements Image Analyzer (version 4.20, Nikon).

To determine the effects of endocytosis inhibitors on OMV uptake, the BMDM were pretreated with methyl-β-cyclodextrin (MβC, 10 mM), cytochalasin D (10 μM), wortmannin (100 nM), filipin (1 μg/ml), amiloride (10 mM), dansylcadaverine (150 μM), or dynasore (80 μM) (Sigma-Aldrich) for 30 min at 37°C. The cells were then exposed for 1 h to OMV (0.5 μg) isolated from the wild-type FSC200 strain or FSC200/Δ*wbtDEF* and thereafter fixed and further processed for microscopy as described above. Non-treated and DMSO-treated cells were used as negative controls. For cell entry quantification, 200 cells from randomly selected fields were evaluated. Results are reported as percentages of the 200 cells analyzed or as the mean of a given dataset.

### Cell Viability, Cytotoxicity Assay, and Apoptotic Assay

To follow the viability and cytotoxic effect of *F. tularensis* FSC200 OMV, the cells were seeded in 96-well tissue culture plates at concentrations of 2 × 10^4^ cells/well for BMDM, 0.5 × 10^4^ cells/well for J774.2, and 0.5 × 10^3^ cells/well for A549 and allowed to adhere overnight. The cell numbers had been optimized by preliminary experiments. The next day, the supernatant was discarded and the cells incubated with OMV (1, 0.5, or 0.05 μg of OMV protein per 1 × 10^4^ cells) in 50 μl of DMEM/well. After 30 min, additional DMEM was added to a final volume of 100 μl/well and the cells were incubated in DMEM at 37°C with 5% CO_2_. BMDM and J774.2 were infected with *F. tularensis* FSC200 at a multiplicity of infection (MOI) of 50 and 100, respectively, for 30 min. The extracellular bacteria were then washed out and the cells further incubated in DMEM. The cell viability was observed in real time using a non-lytic bioluminescence RealTime-Glo^TM^ MT Cell Viability Assay (Promega, Madison, WI, United States). Cytotoxicity was assayed in parallel using fluorescence CellTox^TM^ Green Cytotoxicity Assay (Promega). The luminescent Caspase-Glo 3/7 Assay (Promega) was used for the measurement of the activity of caspase-3/caspase-7. The samples were processed and the luminescence and fluorescence were measured at indicated time points on a FLUOStar OPTIMA plate reader (BMG Labtech, Ortenberg, Germany) according to the manufacturer’s instructions.

### Evaluation of Cytokine Release in Outer Membrane Vesicles-Treated Macrophages

For the evaluation of cytokine release by cytokine arrays, the BMDM seeded on 96-well plates at a concentration of 2 × 10^4^ cells/well were treated with 1 or 0.1 μg OMV in 150 μl DMEM or infected with the FSC200 strain at MOI of 50 in the same manner as that for the cell viability assay (see above). The supernatants were collected after 4 and 24 h of co-incubation and stored at −80°C until needed. The cytokine profiles were analyzed in collected undiluted supernatants using a fluorescence-based multiplex Quantibody ELISA microarray chip (RayBiotech, Norcross, GA, United States) with the following set of screened cytokines: granulocyte colony-stimulating factor (G-CSF), granulocyte–macrophage colony-stimulating factor (GM-CSF), interleukin 1α (IL-1α), IL-1β, IL-2, IL-3, IL-4, IL-5, IL-6, IL-7, IL-9, IL-10, IL-12p70, IL-13, IL-15, IL-17, IL-21, IL-23, IFN-γ, tumor necrosis factor alpha (TNF-α), CXCL-1 (keratinocyte-derive chemokine, KC), monocyte chemoattractant protein 1 (MCP-1), macrophage colony-stimulating factor (M-CSF), RANTES (regulated on activation, normal T-cell expressed, and secreted), and vascular endothelial growth factor (VEGF). The evaluation was performed according to the manufacturer’s protocol. The cytokine concentrations were calculated against the standards using the software H20 OV Q-Analyzer v8.20.4 (RayBiotech). Three replicates of each sample were evaluated for each cytokine in one experiment. The experiments were repeated two or four times, with comparable results.

For confirmation, cytokines that were significantly changed were also quantified by means of classical enzyme-linked immunosorbent assay (ELISA). BMDM at a concentration of 1 × 10^5^ cells/well were adhered on 24-well plates and treated with 200 μl of DMEM containing 0.5 or 5 μg of OMV or infected with FSC200 at MOI of 50. The plates were centrifuged at 400 × *g* for 5 min at RT. After 1 h of incubation, the infected cells were washed twice with PBS to remove the extracellular bacteria and further cultivated in 500 μl of fresh DMEM. To the OMV-treated cells, 300 μl of fresh DMEM was added at the same time. At 4 and 24 h of co-incubation, the cell supernatants were collected and stored at −80°C until needed. The quantities of secreted cytokines/chemokines were measured using mouse ELISA kits against IL-1α, IL-6, IL-10, IL-12p70, TNF-α, GM-CSF, MCP-1, and CXCL-1 (KC) (Thermo Fisher Scientific, Waltham, MA, United States) according to the manufacturer’s instructions. The absorbance was measured at 450 nm on a Paradigm detection platform (Beckman Coulter, Brea, CA, United States). The resulting concentrations were determined from standard calibration curves.

### Stimulated Emission Depletion Super-Resolution Microscopy

For immunofluorescence, the staining protocol was adopted from www.cellsignal.com, with some modifications. Briefly, the differentiated BMDM were attached to no. 1.5 CSHP high-precision cover glasses (Paul Marienfeld, Lauda-Königshofen, Germany) at a concentration of 5 × 10^5^ cells/well in 24-well plates and treated with OMV at a dose of 0.5 μg/1 × 10^5^ cells or infected with FSC200 at MOI of 50, followed by centrifugation at 400 × *g* for 5 min at RT for synchronization. The extracellular bacteria were removed by a thorough wash with PBS (three times). At selected time intervals (1, 6, and 12 h post-treatment), the cells were washed with warm PBS (three times), fixed with 4% PFA for 15 min at RT, and then washed again three times with PBS. After PFA fixation, the cells were permeabilized with ice-cold methanol for 10 min at −20°C. The cells were blocked for 1 h in PBS containing 0.1% saponin (Sigma-Aldrich) with the addition of 2.5% fetal calf serum and 2.5% BSA and then incubated with primary antibodies [mouse anti-*F. tularensis* LPS monoclonal antibody FB11 (Abcam, Cambridge, United Kingdom) and rat anti-MHC class II antibody M5/114.15.2 (BioLegend, San Diego, CA, United States)] and secondary antibodies (goat anti-mouse Alexa 555 and goat anti-rat Alexa 488) for 1 h each consecutively. The coverslips were mounted using 4% *n*-propyl gallate in glycerol. Samples were analyzed with stimulated emission depletion (STED) super-resolution microscopy (Leica TCS SP8 STED 3X, 660-nm CW depletion laser). Image reconstruction was performed using Huygens Professional (SVI) software and post-editing was with Fiji imaging software (Schindelin et al., 2012).

### Transmission Electron Microscopy

#### Examination of *F. tularensis* Interaction With Macrophages

For the morphological analysis of the adherence and phagocytic uptake of *F. tularensis*, we proceeded as previously described, albeit with minor modifications (Clemens et al., 2012; McCaig et al., 2013). Briefly, 6 × 10^6^ of BMDM were pelleted (800 × *g*, 10 min at 4°C) and 1 ml of *F. tularensis* FSC200 bacterial suspension (chilled to 4°C) was added to the pelleted cells at an approximate MOI of 2,000. The cells were centrifuged sequentially at 200 × *g* and 800 × *g*, each for 10 min at 4°C. The supernatant was removed and the tube with cells and bacteria pellets was placed in a 37°C water bath for 5 min and then fixed with 2.5% glutaraldehyde in 0.1 M Na/K phosphate buffer, pH 7.2–7.4 (Sörensen’s buffer, SB). The cells and bacteria were then resuspended in a very small volume of SB prewarmed to 37°C and added to prewarmed 2% low-melting-point agarose in water in a 1:1 ratio. Hardened agarose with biological material was cut into small pieces and post-fixed with 1% osmium tetroxide in SB for 2 h at RT in darkness. Pieces were dehydrated in an ethanol series, and during this process, additionally stained with 1% uranyl acetate in 50% ethanol overnight at 4°C. Dehydrated pieces were embedded in Epon–Durcupan resin. After polymerization for 72 h at 60°C, resin blocks with samples were cut into 80-nm ultrathin sections and collected on 200-mesh size copper grids. The sections were examined in a JEOL JEM-1400Flash transmission electron microscope operated at 80 kV and equipped with a Matataki Flash sCMOS camera (JEOL Ltd., Tokyo, Japan).

For morphological analysis of the later infection stages, 1 × 10^6^ cells/well adhered to the glass coverslips in 24-well plates were infected with bacterial suspension at MOI of 100 and centrifuged at 400 × *g*, for 3 min at RT (time 0). After 1 h of incubation at 37°C in 5% CO_2_, the cells were washed twice with warm PBS and further incubated in fresh DMEM under the same conditions (for a 6-h interval only). At 5 min and 6 h after infection, BMDM were washed with warm SB and fixed with 2.5% glutaraldehyde in SB for 30 min at RT and subsequently overnight at 4°C. Infected cells on glass coverslips were post-fixed with 1% osmium tetroxide in SB for 2 h at RT in darkness and later dehydrated in an ethanol series. During this process, the cells were additionally stained with 1% uranyl acetate in 50% ethanol overnight at 4°C. Afterward, the cells were embedded in Epon–Durcupan resin. After polymerization for 72 h at 60°C, resin blocks with samples were cut into 80-nm ultrathin sections and collected on 200-mesh size copper grids. Sections were examined with the JEOL JEM-1400Flash transmission electron microscope operated at 80 kV equipped with a Matataki Flash sCMOS camera (JEOL Ltd.).

For statistical analysis of the bacterial infection after 5 min, the Limitless Panorama ultrawide area montage system (JEOL Ltd.) was used, which enables capturing a large area of the sample containing 213 cells. The collected image data were manually processed and every cell was visually evaluated with reference to specific criteria. If a cell was infected, the total number of bacteria in its cytoplasm was counted and an evaluation was made on whether these bacteria were encapsulated in phagosome or had direct contact to the cytoplasm. The number of bacteria in each category was counted. Subsequently, each bacterium was evaluated for the presence of OMV. Those with developed OMV were counted and evaluated for whether they were encapsulated in complete phagosome or had direct contact to the cytoplasm.

#### Examination of Macrophage Interaction With Isolated Outer Membrane Vesicles

Bone marrow-derived macrophages (1 × 10^6^ cells/well) adhered to the glass coverslips in 24-well plates were treated with approximately 8 μg of OMV in 200 μl/well of DMEM, centrifuged at 800 × *g* for 5 min at RT, and then incubated at 37°C in 5% CO_2_. After 1 h, 800 μl of warm DMEM was added to each well. At 1, 6, and 24 h, the cells were briefly rinsed with warm SB and fixed in 3% freshly depolymerized PFA with 0.25% glutaraldehyde in SB (1 h at RT). Chemical fixation continued with incubation in 0.02 M glycine in SB for 10 min and dehydration in an ethanol series, all on ice. The samples were embedded in LR White acrylic resin and polymerized for 72 h at 4°C under UV light. Resin blocks with samples were cut into 80-nm ultrathin sections and collected on 200-mesh size copper grids.

For immunolabeling, 80-nm ultrathin sections on 200-mesh gilded grids were first placed for 20 min on 10% normal serum, 0.2% cold water fish skin gelatin in PBS with 0.01% (*v*/*v*) Tween 20 (PBT) containing 1% (*w*/*v*) BSA, and then incubated with anti-LPS primary antibody in 1% normal serum, 0.2% cold water fish skin gelatin in PBT with 1% BSA for 1 h. After a series of washing with PBT, the sections were incubated with secondary goat anti-mouse 12 nm gold-conjugated antibody in 1% normal serum, 0.2% cold water fish skin gelatin in PBT with 1% BSA for 1 h. The sections were then repeatedly washed in PBT and double-distilled water, air dried, and then examined in the JEOL JEM-1400Flash transmission electron microscope operated at 80 kV equipped with a Matataki Flash sCMOS camera (JEOL Ltd.).

### Statistical Analysis

Each experiment was independently repeated at least three times, and the assay was performed in triplicate for each time interval and strain in an experiment. The ELISA experiment was performed in triplicate and repeated twice, with similar results. The Prism 6 program (GraphPad, San Diego, CA, United States) was used for the statistical analysis. The values were expressed as the mean ± standard error of the mean (SEM) and analyzed for significance using ANOVA with a recommended multiple-comparison posttest. Differences were considered statistically significant at *p* < 0.05. Cell viability, cytotoxicity, and caspase-3/caspase-7 activity were expressed as percentages relative to the untreated control.

## Results

### Interaction of *F. tularensis* Outer Membrane Vesicles With Host Cell

#### Outer Membrane Vesicles Enter and Accumulate in Macrophages, but Not Epithelial Cell Line A549

**FIGURE 1:**
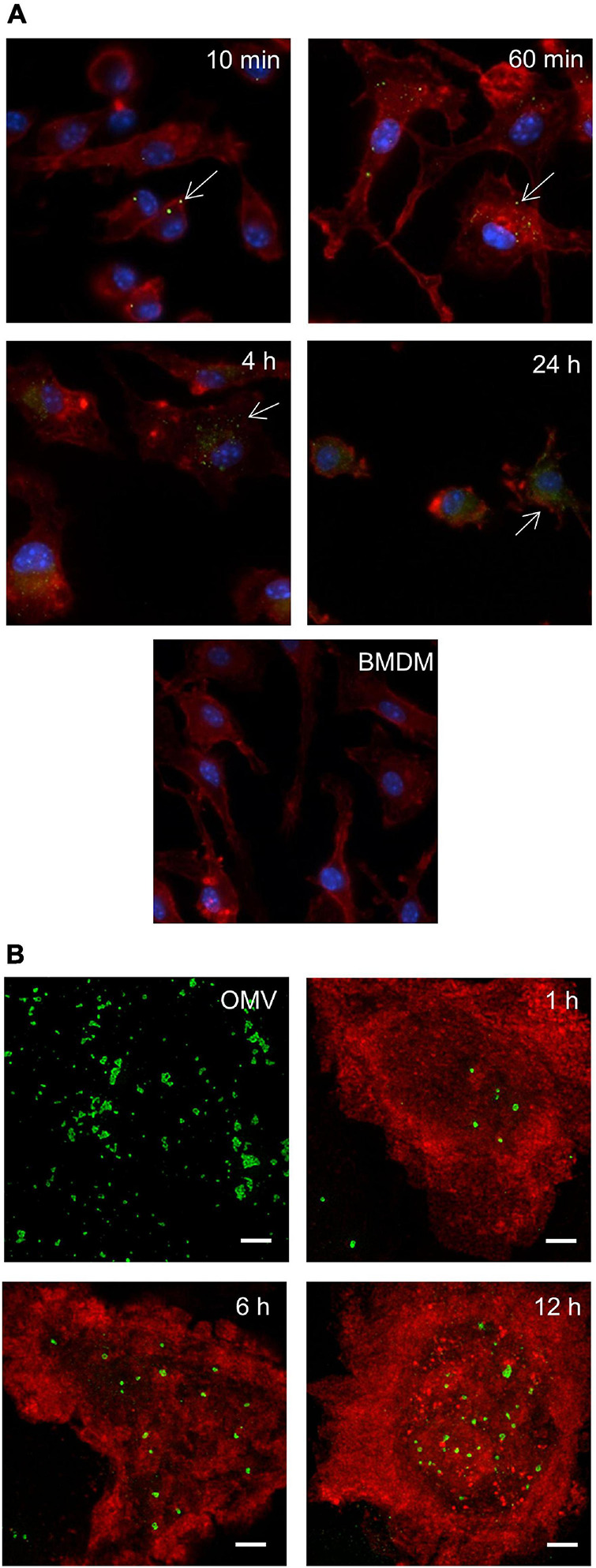
Outer membrane vesicle (OMV) entry into bone marrow-derived macrophages (BMDM) by fluorescence microscopy. BMDM seeded on cover glasses were co-incubated with *Francisella tularensis*-derived OMV (Ft-OMV) for the indicated time, fixed, and immunostained for fluorescence microscopy **(A)** and stimulated emission depletion (STED) microscopy **(B)**. **(A)** Ft-OMV were visualized by immunostaining with purified immune rabbit polyclonal anti-*F. tularensis* serum, followed by Alexa Fluor^TM^ 488 anti-rabbit immunoglobulin G (IgG) (*green* signal). Actin was stained with phalloidin-TRITC (*red* signal) and cell nuclei with DAPI (*blue* signal). Arrows point to the internalized OMV. Untreated BMDM are shown as control. The accumulation of OMV signals around the nucleus is observed in the latest interval. The samples were observed using a ×60 objective. **(B)** Ft-OMV were stained with the anti-*F. tularensis* lipopolysaccharide (LPS) antibody followed by Alexa Fluor^TM^ 555 anti-mouse IgG (*green*) and the BMDM membrane with the anti-MHC class II antibody followed by Alexa Fluor^TM^ 488 anti-rat IgG (*red*). OMV alone are shown on the *upper left*. Shown are the maximal intensity projections of example cells from three independent experiments. Scale bar, 2 μm.

The direct interaction or internalization of OMV derived from pathogenic bacteria has been reported in a number of studies. We therefore decided to first verify the ability of *F. tularensis*-derived OMV (Ft-OMV) to interact with macrophages and epithelial cells. Macrophages are generally regarded as the primary mammalian target cells of *F. tularensis* replication, but this pathogen is also able to occupy non-phagocytic epithelial cells (Hall et al., 2007; Craven et al., 2008; Lo et al., 2013). The isolated OMV were co-incubated with primary bone marrow macrophages, the monocyte–macrophage cell line J774.2, or the lung epithelial cell line A549 for 10 min or for 1, 4, and 24 h. OMV were then visualized using the purified immune rabbit polyclonal anti-*F. tularensis* serum and FITC-labeled secondary antibody and evaluated using fluorescence microscopy. Whereas only sporadic amounts of OMV were detected within the A549 epithelial cells in any of the monitored time intervals (Supplementary Figure 1), the OMV were observed inside the BMDM after only 10 min of co-incubation. Moreover, the amounts of internalized vesicles increased continuously, and at 24 h, the green fluorescence signal indicating OMV presence was accumulated mostly around the cell nucleus (Figure 1A). A similar rate of OMV entry was also observed for the J774.2 cell line. To ensure that OMV really do accumulate inside the host cells and do not stick onto their surfaces, we also confirmed our observation using high-resolution microscopy (STED) (Figure 1B).

#### Outer Membrane Vesicles–Host Cell Interaction Analyzed by Immunoelectron Microscopy

**FIGURE 2:**
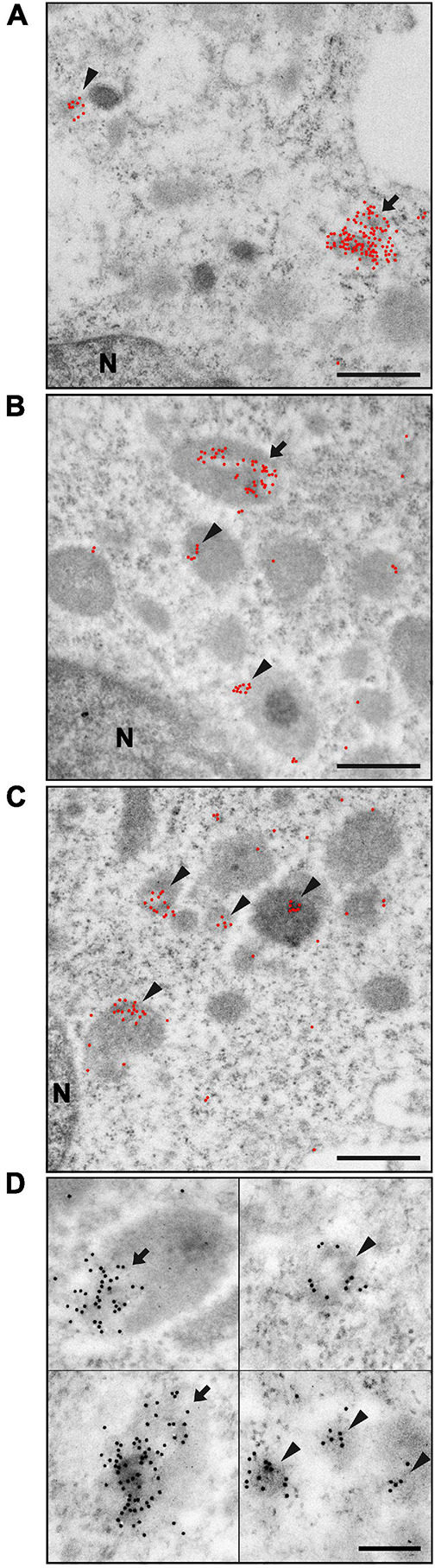
Immunoelectron microscopy of ultrathin sections of bone marrow-derived macrophages (BMDM) exposed to *Francisella tularensis*-derived outer membrane vesicles (Ft-OMV) for 1 h **(A)**, 6 h **(B)**, and 24 h **(C)**. Gold particles are highlighted as *red dots*, small clusters are marked with an *arrowhead*, and large clusters with an *arrow*. **(A)** Immunolabeling after 1 h exposure showed the most significant fraction of large gold particle clusters (more than 20 particles per cluster) associated with darker structures of sizes around 0.5 μm. However, this fraction was still minor when compared with the smaller gold particle clusters. **(B)** After 6 h exposure, the number of large clusters decreased and the number of smaller clusters increased; the total number of clusters increased as well. **(C)** Exposure for 24 h led to a significant increase in the number of small clusters, with the large ones becoming rather rare. The high number of small clusters further increased the total number of clusters. **(D)** Details of the most typical labeled structures irrespective of exposure time. Larger aggregates localized at the periphery of the dark structure (*upper left*) or covering the entire structure (*lower left*). Smaller aggregates found as isolated (*upper right*) or in groups (*lower right*). In the *upper right picture*, typical appearance of the light spot in the darker cellular structure is shown. Scale bar, 500 nm **(A–C)** and 200 nm **(D)**. The original image without highlighted gold particles is in Supplementary Figure 5.

To further analyze the interaction of Ft-OMV with primary macrophages, we utilized immunoelectron microscopy of ultrathin sections of BMDM exposed to Ft-OMV for 1, 6, and 24 h. The OMV were visualized using immunogold labeling with the anti-LPS antibody (Figures 2A–C). Already 1 h after the exposure, multiple clusters of gold particles were observed indicating the position of the anti-LPS antibody binding sites (Figure 2A). The labeling was mostly clustered in groups and connected with some underlying medium dark roundish structures, usually with a light spot surrounded by the labels. Because cellular membranes are commonly not well preserved in samples processed for immunolabeling due to mild fixation and the extractive effect of dehydration and monomeric acrylic resin, it is sometimes difficult unambiguously to define by morphology the type of membrane-bounded structures. Due to this uncertainty, the labeled cellular structures were not exactly identified.

The size of these labeled cellular structures was up to 0.6 μm in diameter. Smaller clusters of labeling (5–20 gold particles) were associated with cellular structures of all sizes, while a majority of larger clusters (greater than 20 gold particles) were found at structures with larger sizes (approx. 0.5 μm). These bigger structures with large gold particle clusters were in all samples in the minority compared to the number of structures labeled with small clusters. Labeling clusters surrounding the light spots were mostly rather localized on the periphery of the structure, but wholly covered structures were found as well (Figure 2D). The larger structures had either a homogenous appearance or revealed a more complex inner structure with dark spots, morphologically resembling late endosomes. These described types of labeled structures were present in all exposed samples. Nevertheless, the overall labeling characteristic changed with the Ft-OMV exposure time. With prolonged time of exposure of the cells to Ft-OMV, the ratio of the smaller to the larger gold particle clusters, as well as the total number of labeled cellular structures, increased. The smaller clusters were often present in groups within samples that had prolonged exposure times (Figures 2C,D). The bigger cellular structures were also labeled at later stages, but the labeling then showed smaller gold particle clusters, while the large clusters still remained in the minority and their numbers did not visibly increase with the exposure time. This overall observation might indicate the accumulation of OMV inside the BMDM over time.

#### Effect of *F. tularensis*-Derived OMV on Host Cell Viability

**FIGURE 3:**
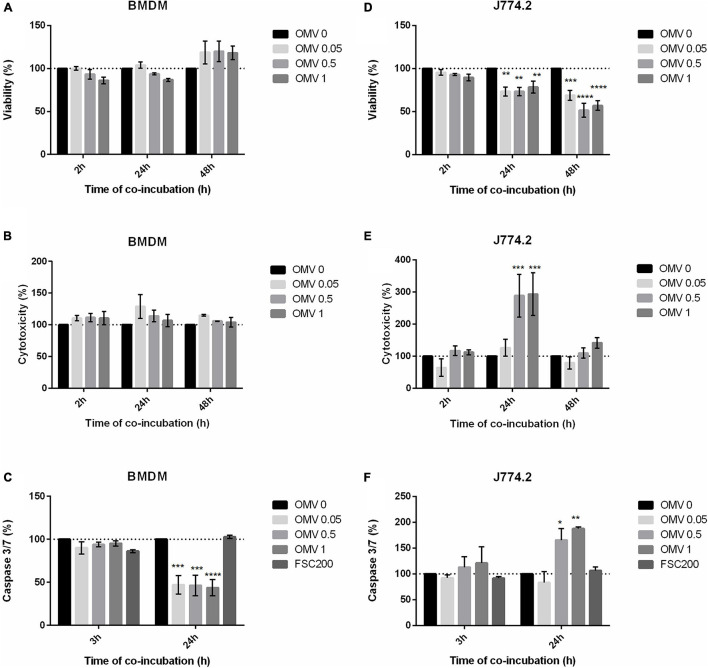
Viability **(A,D)**, induction of cytotoxicity **(B,E)**, and apoptosis **(C,F)** in indicated cells treated with various concentrations of *Francisella tularensis*-derived outer membrane vesicles (Ft-OMV). Control groups: non-treated cells and cells infected with *F. tularensis* FSC200 (for apoptosis only). The values are expressed as percentages relative to the non-treated control (OMV 0) which is highlighted by the dotted line. Experiments were performed in triplicate wells at least three times. Data are the mean ± SEM from three independent experiments. *p < 0.05, **p < 0.01, ***p < 0.001, ****p < 0.0001 (*versus* control); two-way ANOVA with Dunnett’s multiple comparisons test.

As Ft-OMV had previously been shown to contain a number of known virulence factors (McCaig et al., 2013; Klimentova et al., 2019, 2021), we expected that they might have a direct impact on host cells. We therefore examined the effect of isolated Ft-OMV on the viability of host cells by monitoring the cell-reducing potential and changes in the cell membrane integrity. Three various doses of purified Ft-OMV were added to BMDM, J774.2, or A549 cells and the indicated parameters were observed during 48 h of co-incubation. In BMDM and J774.2, we additionally measured the activity of caspase-3 and caspase-7, the primary effector caspases in the apoptosis pathway, 24 h after the co-incubation. In primary BMDM, none of the three applied doses of OMV showed a significant effect on the cell viability and membrane integrity (Figures 3A,B). Interestingly, the activity rates of caspase-3/caspase-7 were significantly decreased in the OMV-treated primary BMDM compared to those of the non-treated cells or cells infected with the virulent FSC200 strain (Figure 3C). On the other hand, the viability of the J774.2 monocyte–macrophage cell line was significantly reduced 24 and 48 h after treatment with Ft-OMV at all dose levels (Figure 3D). After 24 h of incubation, higher doses of Ft-OMV caused a significant disruption of membrane integrity and increased the activity of caspase-3/caspase-7 in J774.2 (Figures 3E,F). No significant changes were observed in the A549 lung epithelial cell line treated with Ft-OMV (Supplementary Figure 2). These results might be related to the reduced ability of Ft-OMV to enter this cell line, as described above.

Taken together, our observations revealed that OMV can have different effects depending on the cell type. While primary macrophages were not significantly affected by the Ft-OMV treatment, significant signs of cytotoxicity were obvious in the macrophage cell line. For subsequent investigations, primary BMDM were selected as the primary host cell model.

#### *F. tularensis* FSC200 Outer Membrane Vesicles Enter Macrophages by Several Uptake Routes

**FIGURE 4:**
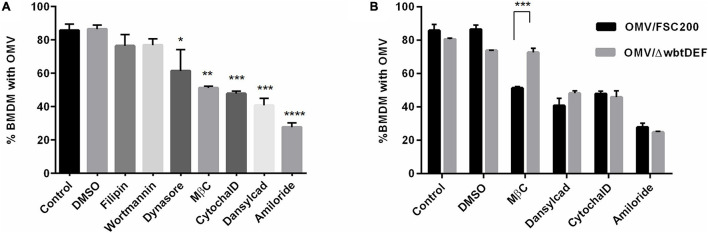
Efficiency of outer membrane vesicle (OMV) uptake by bone marrow-derived macrophages (BMDM) pretreated with the indicated endocytosis inhibitors, DMSO (vehicle control), or untreated (control). **(A)** Pretreated cells were exposed to OMV isolated from the wild-type FSC200 strain for 1 h, and OMV uptake was then expressed as the percentage of cells with at least one internalized OMV from 200 analyzed cells. **(B)** Comparison of the uptake of OMV derived from the Δ*wbtDEF* strain with inactivated O-antigen production. Data are the mean ± SEM from three independent experiments. *p < 0.05, **p < 0.01, ***p < 0.001, ****p < 0.0001; one-way ANOVA with Dunnett’s multiple comparisons test **(A)** or two-way ANOVA with Sidak’s multiple comparisons test **(B)**.

The internalization of OMV in BMDM was further evaluated by treatment with various endocytic inhibitors prior to the addition of OMV. About 86% of non-treated or DMSO-treated cells contained an average of 5 OMV per cell after 1 h of co-incubation. Pretreatment with any of the selected inhibitors resulted in a decreased OMV uptake and decreased number of OMV per cell (Figure 4A). The smallest statistically non-significant effects on OMV uptake were seen with the cholesterol-binding agent filipin (76% of cells, 3 OMV per cell, a 10% reduction in internalization), the phosphatidylinositol kinase inhibitor wortmannin (77%, 2.5 OMV per cell, a 9% reduction), and dynasore, which blocks the activity of dynamin GTPase (61% of cells, 2.5 OMV per cell, a 25% reduction). On the other hand, a significant inhibition of OMV uptake was caused by the cholesterol sequestering agent MβC (51%, 2 OMV per cell, a 40% reduction), the F-actin depolymerization agent cytochalasin D (48%, 1.6 OMV per cell, a 44% reduction), and dansylcadaverine, which inhibits receptor internalization (41%, 1.8 OMV per cell, a 52% reduction). The most pronounced decrease in OMV uptake was evident with amiloride, the inhibitor of macropinocytosis (28%, 1.9 OMV per cell, a 68% reduction in internalization). These data show that OMV are able to access different uptake routes and that macropinocytosis seems to be the preferred internalization pathway. The observed effect of dansylcadaverine indicates the involvement of clathrin-dependent endocytosis. MβC is known to inhibit the internalization of caveolae and lipid raft through the depletion of cholesterol from the cell membrane. It can also perturb clathrin-mediated endocytosis inasmuch as clathrin uptake requires cholesterol as well (Rodal et al., 1999). As we detected only a minor effect of filipin, the Ft-OMV uptake inhibition induced by MβC might also be associated with its ability to disrupt the clathrin-mediated endocytosis. Neither can the possible involvement of another cholesterol-dependent mechanism be fully ruled out. Our observations are not surprising because the OMV of other bacteria have also been reported to be internalized *via* several routes, and this is widely regarded to be a consequence of their size heterogeneity (O’Donoghue and Krachler, 2016; Turner et al., 2018; Jones et al., 2020). As observed in Figure 1B, OMV tend to make clumps, which further contribute to their size heterogeneity. The O-antigen structural region of LPS is known to be critical for OMV entry; therefore, we also tested the uptake of OMV isolated from the *F. tularensis* FSC200 mutant strain with abrogated O-antigen production (FSC200/Δ*wbtDEF*). The only difference, in comparison to the OMV isolated from the O-antigen-positive wild-type strain, was observed in cells pretreated with MβC (Figure 4B). This agent had no obvious effect on the uptake of OMV derived from the O-antigen-deficient strain, thereby indicating that the presence of O-antigen is essential for the cholesterol-dependent endocytic route. Because the uptake of O-antigen-depleted OMV was affected by the inhibitor dansylcadaverine to the same extent as was that of the Ft-OMV with intact O-antigen by the cholesterol-dependent endocytic route, the O-antigen seems not to be responsible for the clathrin-dependent endocytosis. The presence of O-antigen thus seems to be crucial for the cholesterol-dependent endocytosis.

#### Cytokine and Chemokine Release in Macrophages After Outer Membrane Vesicles Treatment

**FIGURE 5:**
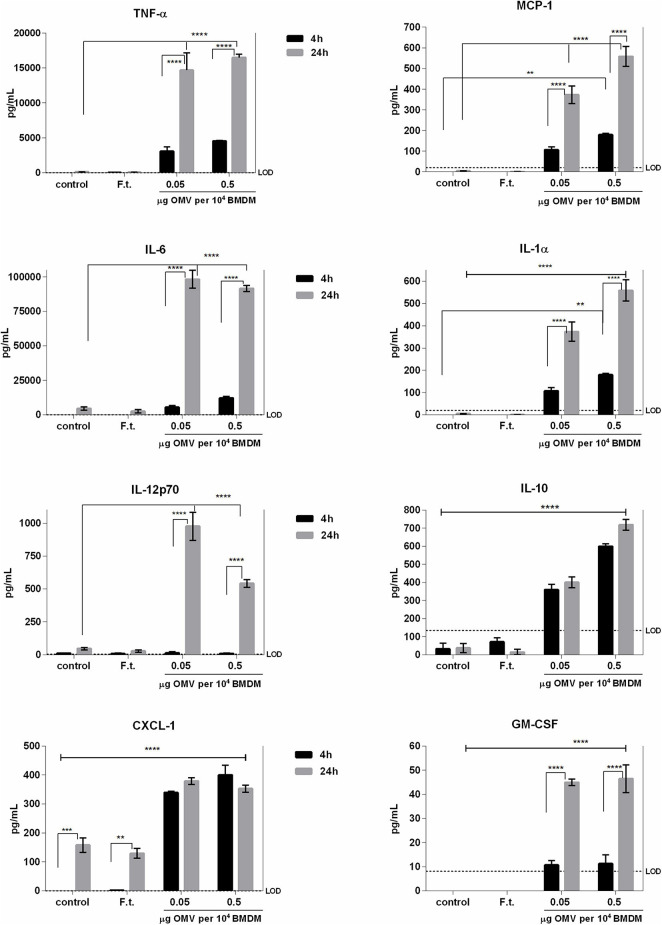
Profiles of cytokine secretion by bone marrow-derived macrophages (BMDM) stimulated with *Francisella tularensis*-derived outer membrane vesicles (Ft-OMV). BMDM were treated with two different doses of Ft-OMV or infected with *F. tularensis* FSC200. Four and 24 h after these stimulations, the cell supernatants were collected and the indicated cytokines were quantified by fluorescence-based multiplex microarray chip ELISA. Data are representative of two to four independent experiments. **p < 0.01, ***p < 0.001, ****p < 0.0001; two-way ANOVA with Tukey’s multiple comparisons test.

We next evaluated the immunomodulatory effects of isolated Ft-OMV on BMDM using the fluorescence-based multiplex microarray chip ELISA. As illustrated in Figure 5, we detected significantly increased levels of TNF-α, IL-6, IL-12p70, CXCL-1 (KC), MCP-1, IL-1α, and IL-10 and slightly increased levels of GM-CSF in the supernatants of BMDM treated with Ft-OMV. For purposes of confirmation, we also quantified the significantly changed cytokines by means of classical ELISA assay (Supplementary Figure 3). The effect of OMV treatment was dose-dependent and increased over time. Inasmuch as the highly induced cytokines are in general regarded as proinflammatory, the Ft-OMV seemed to evoke primarily inflammatory responses in macrophages. In contrast, the infection of BMDM by the whole viable bacterium FSC200 did not induce the release of any of the studied cytokines. This corresponds with the well-described ability of *Francisella* to actively inhibit the innate immune pathways, for which the viability and structural integrity of a bacterium are essential (Telepnev et al., 2003; Singh et al., 2013; Holland et al., 2016; Putzova et al., 2017).

### *F. tularensis*-Derived OMV Production During Infection of Macrophages

**FIGURE 6:**
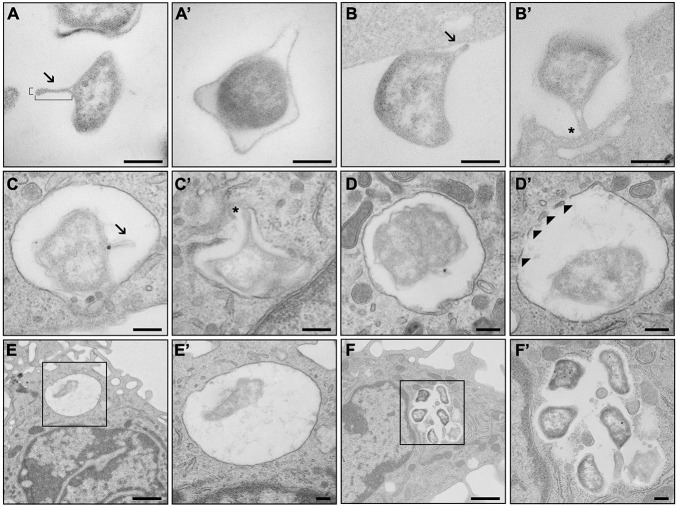
TEM analysis of outer membrane vesicle (OMV) production during infection of macrophages. **(A)** *Francisella tularensis* with developed OMV of standard proportions (180 nm long, 40 nm wide). **(A′)** Protrusions of the outer membrane that are not OMV. **(B)** OMV of *F. tularensis* in direct contact with bone marrow-derived macrophages (BMDM) facing toward the cell. **(B′)** OMV facilitating contact of *F. tularensis* with BMDM. **(C)** *F. tularensis* encapsulated inside a phagosome of BMDM with developed OMV. **(C′)** OMV in connection with the phagosomal membrane. **(D)** *F. tularensis* encapsulated in complete phagosome of BMDM. **(D′)** Decomposition of the phagosomal membrane in the presence of *F. tularensis*. **(E)** Singular *F. tularensis* in complete phagosome 5 min after infection. **(F)** Groups of *F. tularensis* are localized freely to the cytoplasm 6 h after infection. **(E′,F′)** Higher magnification images of black boxes on **(E,F)** respectively. *Arrowheads*, fragments of phagosomal membrane; *arrows*, OMV; *asterisk*, connection of OMV to BMDM. Scale bar, 200 nm and 1 μm **(E,F)**.

The production of OMV in *F. tularensis* has so far been studied in bacteria cultivated extracellularly, and OMV were then isolated from the bacterial culture media (Klimentova et al., 2019, 2021). Here, we also aimed to explore whether this intracellular pathogenic bacterium produces OMV or OMV-like structures when it comes in contact with its primary host cell—the macrophage. For this purpose, we examined *F. tularensis* FSC200 during different stages of infection of BMDM using TEM. The cells were infected with bacteria grown in BHI medium to the early log phase (OD_600_ = 0.3, approx. 4 h of growth), wherein they produced only minimal amounts of vesicles (McCaig et al., 2013). To examine bacterial adherence and uptake, the bacteria at a relatively high MOI (around 2,000 bacteria per cell) were added to BMDM in suspension, fixed after 5 min of co-incubation at 37°C, and then further processed for TEM. This standard procedure should ensure contact with the cell by a sufficient number of bacteria, which is essential for TEM analysis (Clemens et al., 2005). Under these conditions, most of the bacteria were localized outside the host cells, either in contact or in close proximity to the outer side of the cytoplasmic membrane of macrophages. Only rarely were they also seen inside the BMDM (about 5%). The shape of the bacteria was very heterogeneous, but noticeable protrusions from the membrane that might represent the formation of OMV could be distinguished. The main criterion for identifying an OMV-like structure among other membrane protrusions is its distinctively prolonged (100–300 nm long) straight and narrow body with a thin base (20–50 nm wide) and a rounded tip (Figure 6). These OMV-like structures were seen in about 62% of the observed outside bacteria (*n* = 484). Most of these structures (71.4%) faced toward the host cells, and 29% of them were in direct contact with the host cell membrane. The outside bacteria were associated with the macrophages mostly *via* the OMV-like structures (94%). Some of these bacteria (78%) were in contact only *via* these protrusions. In other bacteria (36%), multiple contact points were observed, but at least one of them was *via* these structures. Only 6% of bacteria were associated without the involvement of OMV-like structures. Concerning the bacteria observed inside the BMDM (*n* = 27), around half of them (56%) had also formed such OMV-like structures. Most of these structures (71%) seemed to mediate the contact of bacteria with the host.

For the TEM analysis of the later infection stages, BMDM monolayers on coverslips were infected with bacteria at MOI of 100. Under these experimental conditions, only the bacteria localized inside the cells could be examined. After a 5-min co-incubation interval, about 29% of all the observed cells (*n* = 213) were infected with one or two bacteria (the total number of observed bacteria was 98). Most of the bacteria (82.7%) were found in complete phagosome, with the remainder (17.3%) occurring in a phagosome only partially surrounded by the limiting membrane, thus indicating the phase of phagosomal escape. Only 9.2% of these bacteria (*n* = 9) formed an OMV-like structure, with nearly all of them being localized in complete phagosome (*n* = 8, 88.9%).

The contrasting data relating to the phagosomal stage most likely reflect the distinct experimental conditions applied for the infection of BMDM in suspension *vs*. BMDM in monolayer. For the BMDM in suspension, we were able to observe and evaluate primarily the adherence and uptake of bacteria and the very early phagosomal stage in only a few infected cells. On the other hand, only the phagosomal stage could be examined in monolayered BMDM infected for the same time interval (5 min). Nevertheless, in both situations, a significantly lower production of OMV-like structures was obvious in bacteria localized within a phagosome.

By 6 h post-infection, almost every cell contained a large number of bacteria. The bacteria were localized free in the cytoplasm, but they gathered in large groups mainly close to the cellular membrane. The outer membrane of the bacteria was wavier, with numerous smaller bulges all around their peripheral membrane, but no typical OMV-like structures could be distinguished.

We also attempted to visualize OMV production from intracellular bacteria 1, 6, and 12 h post-infection using the super-resolution fluorescence microscopy (STED), but no release of OMV-like structures was observed using this approach (Supplementary Figure 4).

## Discussion

The roles of OMV derived from pathogenic bacteria in relation to host cells have been demonstrated in a number of studies (Cai et al., 2018; Gao and van der Veen, 2020), and it is now widely accepted that they may contribute significantly to bacterial pathogenesis and host–pathogen interaction. Depending on the original bacterial agent and the host cell type, these structures are able to involve themselves in a diverse set of biological functions, and their final roles can be either defensive or offensive (MacDonald and Kuehn, 2012; Avila-Calderón et al., 2014; Jan, 2017). They might reinforce bacterial adhesion and invasion through the host tissues (Elmi et al., 2016; Metruccio et al., 2016). In the host cell, OMV are usually reported to elicit inflammatory responses by the induction of relevant cytokines and chemokines (Kaparakis-Liaskos and Ferrero, 2015). On the other hand, some OMV have been shown to limit inflammation and the viability of immune cells, allowing the bacteria to escape the host defense mechanisms and to proliferate imperceptibly (Chmiela et al., 2018). Many pathogenic bacteria have been reported to use OMV to transfer virulence factors to the host cells, even over a distance within the host. This has been reported not only for extracellular but also for intracellular bacteria. For example, *S. typhimurium* localized inside the *Salmonella*-containing vacuole produces OMV that can deliver genotoxin to the nuclei of bystander cells (Guidi et al., 2013). Extracellular vesicles released from *Mycobacterium tuberculosis* transfer immunologically active glycolipids to T cells (Athman et al., 2015). On the other hand, *Listeria monocytogenes* release membrane vesicles intracellularly to control the lytic activity of listeriolysin, which promotes the bacteria to survive inside the host cell (Vdovikova et al., 2017). In other intracellular bacteria, the OMV have also been established to modulate cellular processes to favor their internalization and persistence in the host cell. OMV from *Brucella abortus* have been shown to promote bacterial internalization by human monocytes and also to downregulate the innate immune response of these cells to *Brucella* infection (Pollak et al., 2012). The OMV released by *L. pneumophila* into phagosomes are able to inhibit the phagosome–lysosome fusion (Jung et al., 2016).

The production of OMV had also been demonstrated in *Francisella* spp., but data regarding the effect on host cells remain so far very limited and moreover come from studies performed on OMV derived from non-pathogenic *F. novicida* (Pierson et al., 2011; McCaig et al., 2013). Although closely related genetically to *F. tularensis*, *F. novicida* is a separate species with significant differences in its pathogenesis and virulence (Kingry and Petersen, 2014). Pathogenic or non-pathogenic bacterial OMV may, however, show great discrepancies in their biological activity (Behrouzi et al., 2018). Here, we report that, even as OMV derived from the fully virulent *F. tularensis* subsp. *holarctica* strain FSC200 can be internalized into the macrophages, their association with A549 lung epithelial cells was, in our experimental conditions, almost negligible. Consistent with this observation, the viability and membrane integrity of the latter cell type was not affected by the Ft-OMV. Interestingly, Pierson et al. (2011) observed that some OMV from *F. novicida* attached to the A549 cell line, but their internalization also could not be clearly demonstrated. Nevertheless, it should be noted that the *F. novicida* OMV analyzed in their study were prepared from the distinct growth phase of bacteria grown in medium of a different composition. Under these conditions, they obtained small spherical vesicles, in contrast to the large tube-shaped ones characterized by McCaig et al. (2013) in *F. novicida* and by our group in *F. tularensis* (Klimentova et al., 2019). Most of the studies focused on OMV–host cell interaction have demonstrated the internalization of the vesicles into epithelial cells (Kunsmann et al., 2015; Mondal et al., 2016; Bielaszewska et al., 2017; O’Donoghue et al., 2017; Turner et al., 2018). Only a few studies have provided direct evidence for OMV uptake into macrophages (Pollak et al., 2012; Vanaja et al., 2016; Hu et al., 2020). This might mainly be due to the fact that, as professional phagocytes, macrophages are naturally programmed to engulf and eliminate any foreign particles, and therefore the internalization of OMV is highly assumed. *Francisella* proliferates primarily in mononuclear phagocytes (macrophages and dendritic cells), but it is also able to infect non-phagocytic cells (epithelial cells, endothelial cells, and hepatocytes). Such entry is less efficient than that in macrophages (Hall et al., 2007), indicating distinct but so far unknown uptake mechanisms. Whether this may also be related to the observed differences in the association of OMV with macrophages and lung epithelial cells remains a subject for discussion and further research.

Several endocytic uptake routes have been implicated in mediating OMV entry into non-phagocytic cells depending on their origin, size, and cargo (O’Donoghue and Krachler, 2016; Turner et al., 2018). On the other hand, data on the exact mechanism of OMV entry into macrophages are quite sparse. Macrophages are characterized by an exceptionally high endocytic activity. Larger particles (>100 nm) are engulfed by phagocytosis and macropinocytosis and smaller ones by pinocytosis, such as clathrin- and non-clathrin-mediated endocytosis. Our data suggest that the Ft-OMV uptake depends primarily on macropinocytosis, then on clathrin-dependent endocytosis, and, to a lesser extent, probably also on clathrin-independent uptake mechanisms. As the OMV produced by *Francisella* are heterogeneous in shape and size (McCaig et al., 2013; Sampath et al., 2018; Klimentova et al., 2019), the involvement of several distinct uptake routes is not so surprising. Whereas macropinocytosis is a non-specific process, the internalization by clathrin-dependent endocytosis is mediated by cell surface receptors. This means that only receptor-specific substances can utilize this pathway. Macropinocytosis is more probably used by larger particles and the OMV clumps. Several previous studies have demonstrated a role of the LPS O-antigen in OMV entry. In *Escherichia coli*, the presence of the LPS O-antigen increases the entry efficiency of OMV into epithelial host cells. OMV lacking the O-antigen require protein receptors for uptake and use of the clathrin-mediated endocytosis as a main route of entry. In contrast, OMV with intact O-antigen enter host cells by the faster and thus more efficient raft-mediated endocytosis (O’Donoghue et al., 2017). Vanaja et al. (2016) demonstrated the ability of *E. coli* OMV to enter macrophages by clathrin-mediated endocytosis, followed by the release of LPS into the cytosol. Based on the data provided in this study, the *F. tularensis* LPS O-antigen seems not to be responsible for the clathrin-mediated endocytosis, but it did contribute to the uptake of Ft-OMV mediated by the cholesterol-dependent route.

Inside the macrophages, the OMV tend to accumulate in cytoplasmic structures and to reside there for at least 24 h. The OMV thus seem able to evade the classical endosomal degradation mechanisms of macrophages. Unfortunately, we were not able to determine the precise origin of these structures. Some of them seemed to resemble late endosomes, but the electron microscopy approach used in our study did not allow further specification. Hence, the elucidation of the intracellular trafficking of isolated OMV and the mechanisms that allow such long persistence inside a professional phagocyte require further extensive studies.

The OMV produced by pathogenic bacteria are loaded with plenty of biologically active substances, such as toxins, LPS, and other virulence factors. Based on the nature of their content, they can induce distinct host cell responses. In some bacteria, the cytotoxic effect of OMV was shown to be due to the presence of LPS or OmpA (Jin et al., 2011; Vanaja et al., 2016). *Francisella* does not produce any yet known toxin, and its LPS has only low toxicity and immunogenicity. Here, we found that the Ft-OMV had no cytotoxic effect and did not influence the viability of primary murine macrophages. Minor cytotoxicity was reported also for *F. novicida* OMV (McCaig et al., 2013). In contrast, the viability of the murine macrophage-like cell line J774A.2 was negatively affected by the Ft-OMV. The harmful effect of *F. novicida* OMV on this cell line was also reported by Pierson et al. (2011). It is thus obvious that the use of different cell types and primary cells *vs*. cell lines may lead to significant disparities in the results. Cautious interpretation is therefore highly advised. Despite their zero toxicity, the Ft-OMV elicited a significant dose-dependent release of a number of proinflammatory cytokines in the primary murine macrophages. On the contrary, the whole bacterium revealed an immunosuppressive effect on these cells, and this has also been described by others (Bauler et al., 2014; Gillette et al., 2014; Fabrik et al., 2018). It is a part of the bacterial strategy to invade the very immune system surveillance that would otherwise activate cellular mechanisms directed to bacterial elimination. In this context, however, the diametric difference between the huge amounts of isolated vesicles used for the BMDM treatment in the presented experiments in comparison with the orders of magnitude lower amount that the bacterium releases during host cell entry should be taken into account. For *Francisella*, it is not clear which particular components of the OMV are responsible for their high immunostimulatory effect. McCaig et al. (2013) described in OMV derived from *F. novicida* that the surface-exposed OMV proteins are only partially responsible for this effect because the proinflammatory effect of OMV was not altered after proteinase K treatment, and it was decreased by heat treatment of OMV.

The proinflammatory effect of Ft-OMV—in contrast to the immunosuppressive effect of the whole bacterium—may appear to be irrelevant during the infection. This raises the questions whether and in which phase of its intracellular life cycle *Francisella* produces OMV. Based on the results of the TEM analysis, it seems that the Ft-OMV or the OMV-like protrusions from the outer membrane might play a role in the contact of the bacterium with the host cell and maybe for the bacterial uptake. The number of observed OMV-like protrusions decreased markedly in the bacteria localized inside the phagosome, and no structures that would resemble the OMV were visible in the bacteria within the host cell cytosol. The production of OMV by *F. novicida* during the initial infection phase has also been reported (McCaig et al., 2013). These authors, however, analyzed only the very early phase of bacterial infection, and in contrast to the fully virulent *F. tularensis*, *F. novicida* lacks the ability to induce immunosuppression (Kingry and Petersen, 2014).

To summarize, in the present study, we provide the first deeper insights into the effects of OMV isolated from the fully virulent *F. tularensis* strain on host cells. OMV exert various effects especially on macrophages, whereas their association with non-phagocytic cells appears to be of minor importance. The Ft-OMV enter the macrophages by various uptake routes and accumulate in specific cytosolic structures for at least 24 h. Their negligible cytotoxicity together with their immunostimulatory effect on these cells might point to future research into their potential as a vaccine candidate. Further experimental evaluation of the OMV–host cell interaction is nevertheless still needed. Moreover, we have demonstrated that *F. tularensis* seems to utilize OMV primarily for the initial contact with the host cells, but their exact role in this process warrants further investigations. Furthermore, the processes of OMV isolation and purification suffer from low yields that are far below the requirements for vaccine development; thus, we propose that future research on *Francisella*-derived OMV considered for vaccine development should not focus solely on naturally secreted vesicles.

## Data Availability Statement

The original contributions presented in the study are included in the article/Supplementary Material, further inquiries can be directed to the corresponding author.

## Ethics Statement

All experiments on mice were conducted under supervision of the Institution’s Animal Unit and were approved by the Animal Care and Use Committee of the Faculty of Military Health Sciences, University of Defence, Hradec Kralove under project number 26/16 (50-29/2016-684800).

## Author Contributions

IP, JK, and JS conceived and designed the study. IP, JK, JB, LH, KK, EV, HR, VF, and OB performed the experiments. IP and JK wrote the manuscript. JS edited the manuscript. All authors contributed to the article and approved the submitted version.

## Conflict of Interest

The authors declare that the research was conducted in the absence of any commercial or financial relationships that could be construed as a potential conflict of interest.

## Publisher’s Note

All claims expressed in this article are solely those of the authors and do not necessarily represent those of their affiliated organizations, or those of the publisher, the editors and the reviewers. Any product that may be evaluated in this article, or claim that may be made by its manufacturer, is not guaranteed or endorsed by the publisher.

## Supplementary Material

Supplementary Figure 1 Fluorescence microscopy of lung epithelial cell line A549 co-incubated with Ft-OMV.

Supplementary Figure 2 Viability and induction of cytotoxicity in lung epithelial cell line A549 treated with various concentrations of Ft-OMV.

Supplementary Figure 3 Confirmation of quantification of the cytokines significantly released from BMDM after OMV treatment by classical ELISA assay.

Supplementary Figure 4 Kinetics of *F. tularensis* internalization into BMDM by STED microscopy.

Supplementary Figure 5 Immunoelectron microscopy of ultrathin sections of BMDM exposed to Ft-OMV, original TEM image.

## Abbreviations

OMV: outer membrane vesicles
Ft-OMV: *F. tularensis*-derived OMV
LPS: lipopolysaccharide
BHI: brain heart infusion
OD: optical density
DMEM: Dulbecco’s modified Eagle’s medium
FBS: fetal bovine serum
BMDM: bone marrow-derived macrophages
PFA: paraformaldehyde
PBS: phosphate-buffered saline
MβC: methyl-β-cyclodextrin
MOI: multiplicity of infection
STED: stimulated emission depletion
SB: Sörensen’s buffer
PBT: phosphate-buffered saline with 0.01% Tween 20
SEM: standard error of the mean
TEM: transmission electron microscopy.
