# Supplementary material for: Francisella tularensis Outer Membrane Vesicles Participate in the Early Phase of Interaction With Macrophages
Source: Front Microbiol. 2021 Oct 15;12:748706. doi: 10.3389/fmicb.2021.748706 (PMC8554293; doi:10.3389/fmicb.2021.748706)
Supplement: Supplementary Figure 1 — Fluorescence microscopy of lung epithelial cell line A549 co-incubated with Ft-OMV. [file Image_1.PDF]

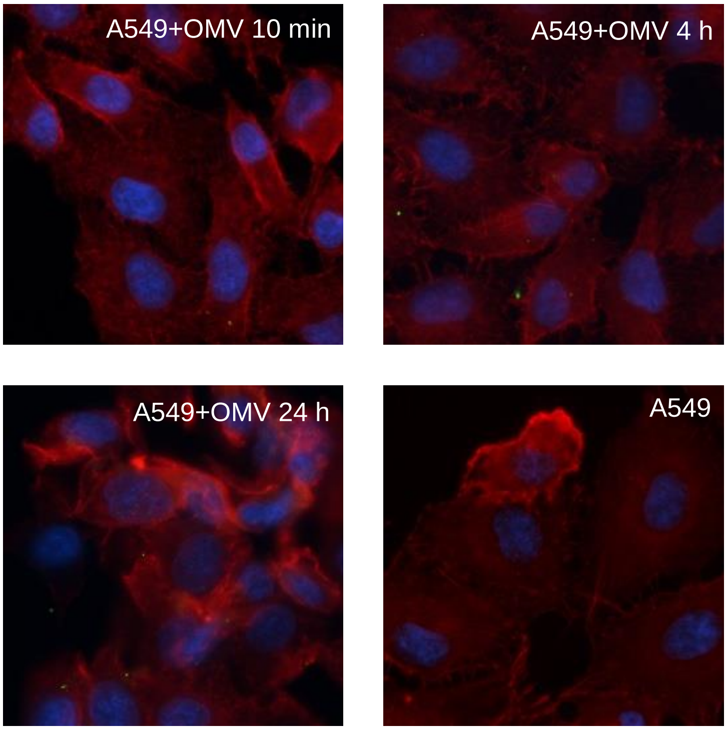

Supplementary Figure 1: Fluorescence microscopy of lung epithelial cell line A549 co-incubated with Ft-OMV for 10 min, 4 and 24 h at 37 °C. The Ft-OMV were visualized by immunostaining with purified immune rabbit polyclonal anti-*F. tularensis* serum, followed by AlexFluor™ 488 anti-rabbit IgG (green signal), actin was stained with Phalloidin-TRITC (red signal) and cell nuclei with DAPI (blue signal). Untreated A549 cells are shown as control. The samples were observed using the 60x objective.
