# Supplementary material for: Francisella tularensis Outer Membrane Vesicles Participate in the Early Phase of Interaction With Macrophages
Source: Front Microbiol. 2021 Oct 15;12:748706. doi: 10.3389/fmicb.2021.748706 (PMC8554293; doi:10.3389/fmicb.2021.748706)
Supplement: Supplementary Figure 2 — Viability and induction of cytotoxicity in lung epithelial cell line A549 treated with various concentrations of Ft-OMV. [file Image_2.PDF]

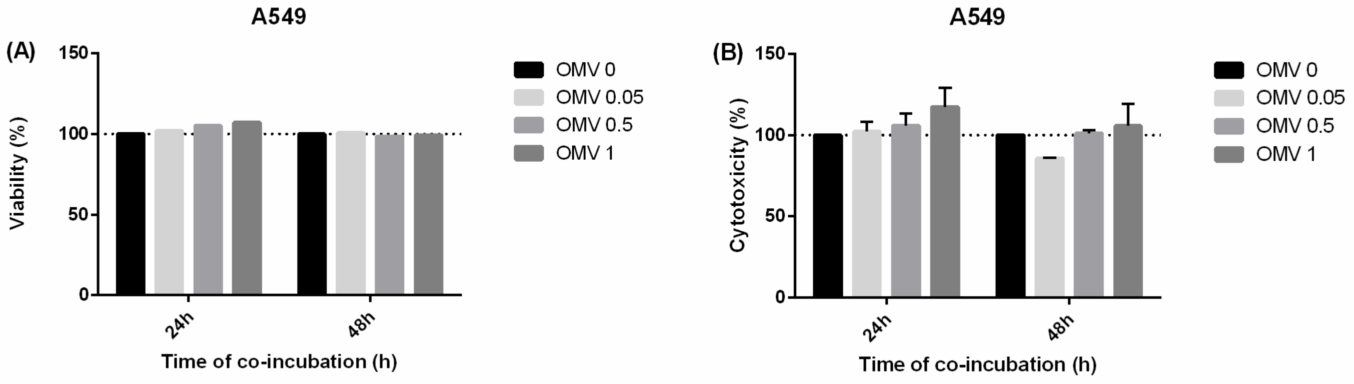

Supplementary Figure 2: Viability (**A**) and induction of cytotoxicity (**B**) in lung epithelial cell line A549 treated with various concentrations of Ft-OMV. Control groups are non-treated-cells. The experiments were performed in triplicate wells at least three times. Data are means ± SEM from three independent experiments. Differences are not significant versus control, two-way ANOVA with Dunnett's multiple comparisons test.
