# Supplementary material for: Francisella tularensis Outer Membrane Vesicles Participate in the Early Phase of Interaction With Macrophages
Source: Front Microbiol. 2021 Oct 15;12:748706. doi: 10.3389/fmicb.2021.748706 (PMC8554293; doi:10.3389/fmicb.2021.748706)
Supplement: Supplementary Figure 3 — Confirmation of quantification of the cytokines significantly released from BMDM after OMV treatment by classical ELISA assay. [file Image_3.PDF]

Supplementary Figure 3: Confirmation of quantification of the significantly released cytokines after OMV treatment by classical ELISA assay. BMDM were treated by different doses of OMV or infected by *F. tularensis* subsp. *holarctica* FSC200 (MOI=50). The supernatants were collected 4, or 24 h post treatment for secreted cytokine quantification by ELISA. Experiments were performed in triplicates, each experiment was repeated twice with comparable results. Data are means ± SEM. * $P < 0.05$, ** $P < 0.01$, *** $P < 0.001$, **** $P < 0.0001$; two-way ANOVA with Tukey's multiple comparisons test.

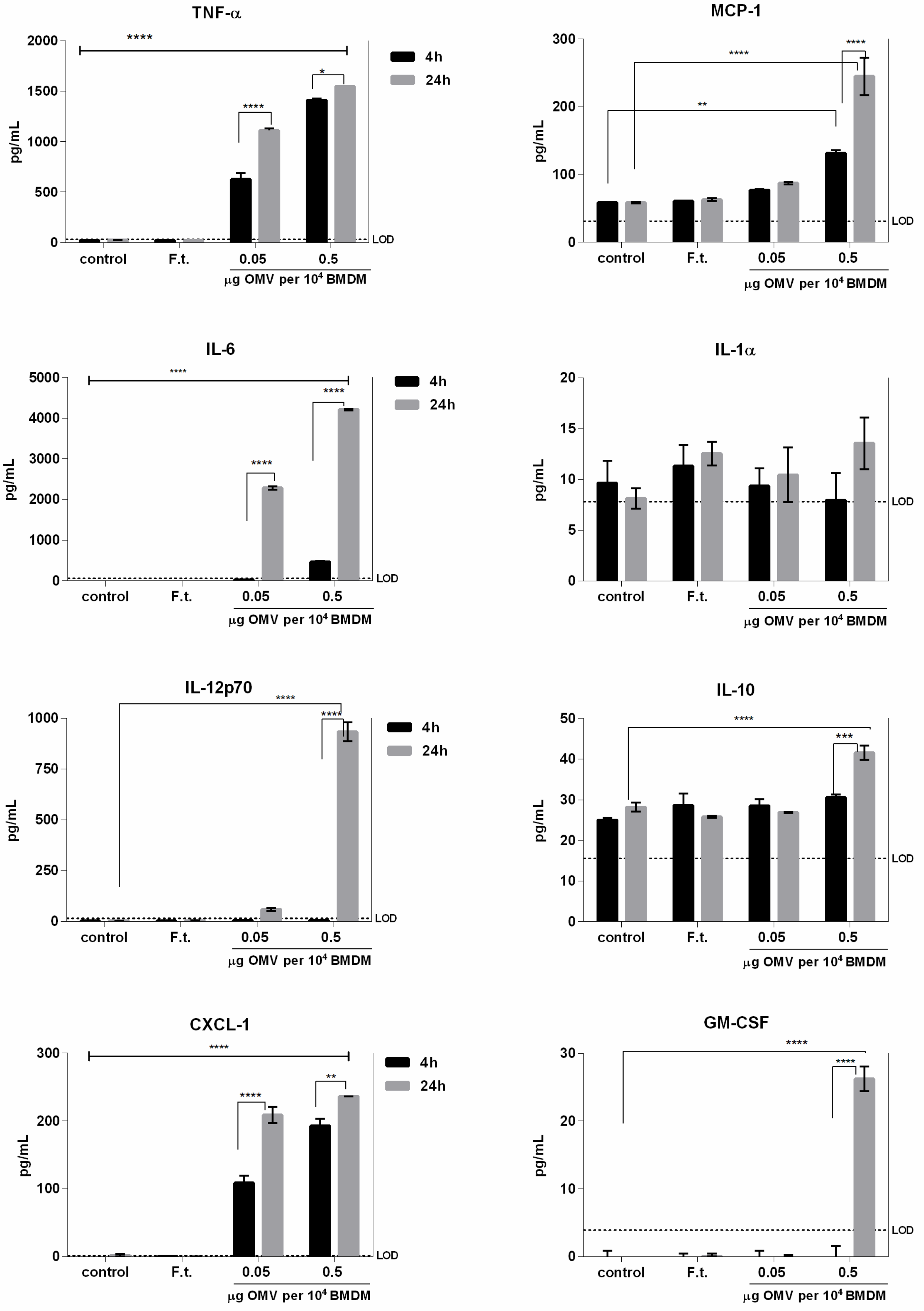
TNF-α
MCP-1
IL-6
IL-1α
IL-12p70
IL-10
CXCL-1
GM-CSF
pg/mL
4h
24h
LOD
control
F.t.
0.05
0.5
μg OMV per 10⁴ BMDM
