# Supplementary material for: Francisella tularensis Outer Membrane Vesicles Participate in the Early Phase of Interaction With Macrophages
Source: Front Microbiol. 2021 Oct 15;12:748706. doi: 10.3389/fmicb.2021.748706 (PMC8554293; doi:10.3389/fmicb.2021.748706)
Supplement: Supplementary Figure 4 — Kinetics of F. tularensis internalization into BMDM by STED microscopy. [file Image_4.PDF]

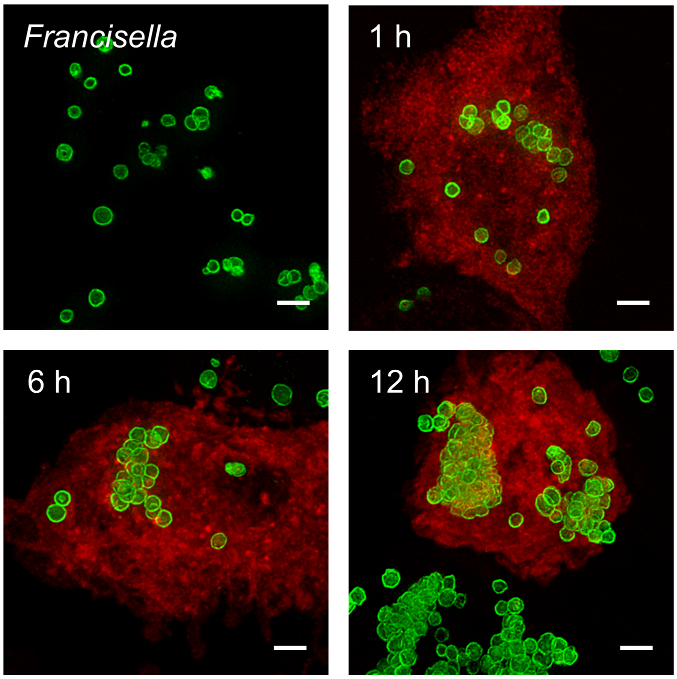

Supplementary Figure 4: Kinetics of *F. tularensis* internalization into BMDM. BMDM seeded on cover glasses and incubated with *F. tularensis* FSC200 for indicated time, or bacteria alone, were fixed and stained for STED microscopy with anti-*F. tularensis* LPS antibody (green) and with anti-MHC class II antibody (red). Maximal intensity projection of example cells from three independent experiments are shown. The scale bar represents length of 2 μm.
