# Supplementary material for: Francisella tularensis Outer Membrane Vesicles Participate in the Early Phase of Interaction With Macrophages
Source: Front Microbiol. 2021 Oct 15;12:748706. doi: 10.3389/fmicb.2021.748706 (PMC8554293; doi:10.3389/fmicb.2021.748706)
Supplement: Supplementary Figure 5 — Immunoelectron microscopy of ultrathin sections of BMDM exposed to Ft-OMV, original TEM image. [file Image_5.PDF]

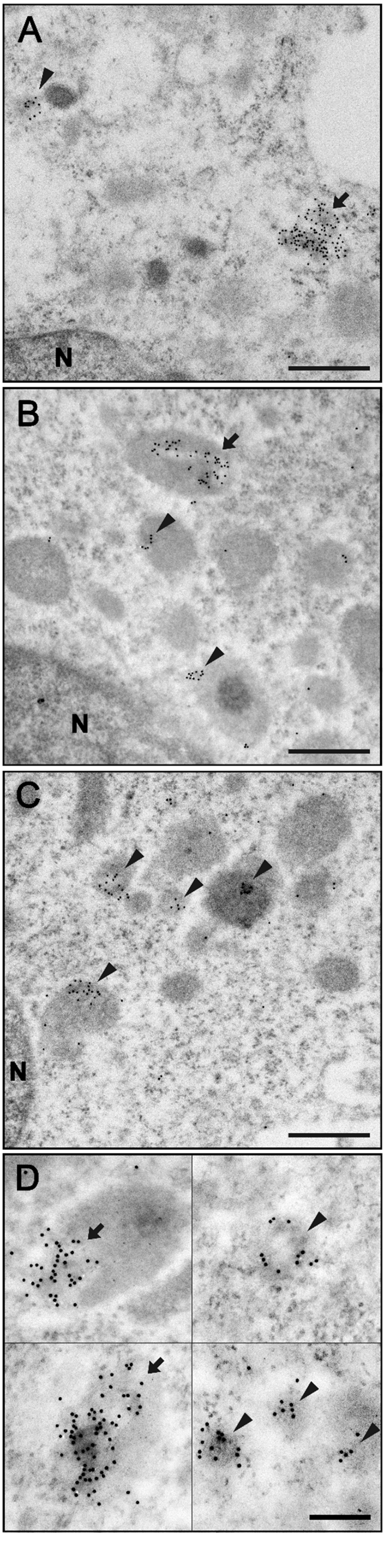

Supplementary Figure 5: Immunoelectron microscopy of ultrathin sections of BMDM exposed to Ft-OMV. (**A** – **C**) Changing character of immunolabeling with increasing exposure time: (**A**) 1 hour, (**B**) 6 hours, and (**C**) 24 hours. (**D**) Details of the most typical labeled structures, irrespective to the exposure time. Smaller clusters are marked with an arrowhead, larger clusters with an arrow. Scale bar in A-C: 500 nm; F: 200 nm.
